# The Role of RAB GTPases and Its Potential in Predicting Immunotherapy Response and Prognosis in Colorectal Cancer

**DOI:** 10.3389/fgene.2022.828373

**Published:** 2022-01-28

**Authors:** Xuefei Jiang, Lanlan Yang, Qianling Gao, Yiting Liu, Xingzhi Feng, Shubiao Ye, Zihuan Yang

**Affiliations:** Guangdong Provincial Key Laboratory of Colorectal and Pelvic Floor Diseases, Guangdong Institute of Gastroenterology, The Sixth Affiliated Hospital of Sun Yat-sen University, Guangzhou, China

**Keywords:** RABs, RAB17, RAB34, colorectal cancer, immune, cell cycle

## Abstract

**Background:** Colorectal cancer (CRC) is the third most common cancer worldwide, in which aberrant activation of the RAS signaling pathway appears frequently. RAB proteins (RABs) are the largest Ras small GTPases superfamily that regulates intracellular membrane trafficking pathways. The dysregulation of RABs have been found in various diseases including cancers. Compared with other members of Ras families, the roles of RABs in colorectal cancer are less well understood.

**Methods:** We analyzed the differential expression and clinicopathological association of RABs in CRC using RNA sequencing and genotyping datasets from TCGA samples. Moreover, the biological function of RAB17 and RAB34 were investigated in CRC cell lines and patient samples.

**Results:** Of the 62 RABs we analyzed in CRC, seven (RAB10, RAB11A, RAB15, RAB17, RAB19, RAB20, and RAB25) were significantly upregulated, while six (RAB6B, RAB9B, RAB12, RAB23, RAB31, and RAB34) were significantly downregulated in tumor tissues as compared to normal. We found that the upregulated-RABs, which were highly expressed in metabolic activated CRC subtype (CMS3), are associated with cell cycle related pathways enrichment and positively correlated with the mismatch repair (MMR) genes in CRC, implying their role in regulating cell metabolism and tumor growth. While, high expression of the downregulated-RABs were significantly associated with poor prognostic CRC mesenchymal subtypes (CMS4), immune checkpoint genes, and tumor infiltrating immune cells, indicating their role in predicting prognosis and immunotherapy efficacy. Interestingly, though RAB34 mRNA is downregulated in CRC, its high expression is significantly associated with poor prognosis. *In vitro* experiments showed that RAB17 overexpression can promote cell proliferation via cell cycle regulation. While, RAB34 overexpression can promote cell migration and invasion and is associated with PD-L1/PD-L2 expression increase in CRC cells.

**Conclusions:** Our study showed that RABs may play important roles in regulating cell cycle and immune-related pathways, therefore might be potential biomarkers in predicting prognosis and immunotherapy response in CRC.

## Introduction

Colorectal cancer (CRC) is the third most prevalent cancer worldwide (Siegel et al., 2020; Siegel et al., 2021). More than 30% of CRC are driven by mutations of the RAS family of genes (Crockett and Nagtegaal, 2019; Du F. et al., 2020; Harada and Morlote, 2020). Oncogenic RAS mutations result in abnormal activation of the EGFR signaling pathway, hence facilitate tumor growth, progression and immune evasion (Sanchez-Vega et al., 2018; Kerk et al., 2021). Targeting Ras-mutant CRC remains a grand challenge to clinical treatment. It is important to better explore the function and mechanism of the RAS family proteins, complexes of RAS proteins with effector and regulators, pathways that are enriched in cancer cells.

The Ras-associated binding proteins (RABs) comprise the largest family of Ras small guanosine triphosphatases (GTPases) that cycle between an inactive guanosine diphosphate (GDP)-bound state and an active guanosine triphosphate (GTP)-bound state. RABs are the identified to be involved in the regulation of vesicular trafficking, including vesicle formation, transportation, membrane docking, and fusion (Pfeffer, 2017; Guerra and Bucci, 2019). In particular, RABs prescribe the directionality of membrane-bound cargo traffic to ensure that the cargo is delivered to the correct destination (Zhen and Stenmark, 2015). Presently, more than 60 RABs have been identified in humans (Anand et al., 2020). Deregulation of RABs has been reported in several diseases, including various cancers (Sun et al., 2018; Ganga et al., 2021). They function either as an oncogene or tumor suppressors depending on cancer types. Nevertheless, the roles of RABs in colorectal cancer are less-well understood.

In this study, we screened multiple genomic datasets for RABs that were differentially expressed between normal and tumor tissues, and systematically evaluated the role of RABs in CRC, including their association with CRC molecular subtypes, immune response, and prognosis. Moreover, we assessed the functional mechanisms of RAB17 and RAB34 in CRC cells.

## Materials and Methods

### Patients and Sample Collection

This study was approved by the ethics committee of the Sixth Affiliated Hospital of Sun Yat-sen University. All patients provided full consent for the study. A total of 4 cases pairs of tumor and adjacent normal tissues (5 cm away from the tumor border) from CRC patients analyzed in this study were provided from the Tissue Bank, Sixth Affiliated Hospital of Sun Yat-sen University, China.

### Plasmid Construction and Transfection

The pcDNA 3.1 (+) plasmid was purchased from Invitrogen. The overexpression plasmids were carried out by inserting the RAB17 and RAB34 sequence into the pcDNA3.1+ plasmid at multiple cloning sites with HindIII restriction enzymes (New England Biolabs, CA, United States) and In-Fusion HD Cloning Kit (Clontech, CA, United States). The overexpression plasmids were transfected into CRC cells with Lipofectamine 3000 and p3000 (Invitrogen, MA, United States) according to the manufacturer’s protocol.

### Cell Culture

CRC cell lines were purchased from the American Type Culture Collection (ATCC, VA, United States). HCT116 and SW480 cells were maintained in RPMI-1640 (GIBCO, NY, United States), supplemented with 10% (v/v) fetal bovine serum (FBS, GIBCO, NY, United States). Cells were allowed to grow in a humidified incubator with 5% CO_2_ at 37°C.

### Western Blot Assays

Protein was extracted by T-PER tissue protein extraction reagent (Thermo, Rockford, IL) with protease inhibitor cocktail set III and phosphatase inhibitor cocktail set II (Millipore, Germany) according to the manufacturer’s protocol. Primary antibodies included GAPDH as loading controls (Proteintech, #60004-1-Ig), RAB17 (Proteintech, #17501-1-AP), RAB34 (SANTA, #SC376898), HA-tag (CST, #3724), CDK2 (CST, #2546), CyclinB1 (Proteintech, 55004-1-AP), PD-L1 (CST, #13684), PD-L2 (Abcam, #ab187662).

### Cell Proliferation, Migration and Invasion Assay

Cell proliferation was constructed with the Incucyte ZOOM device (ESSEN bioscience, United States). 5000 cells/well were seeded into a 96-well plate and automatically monitored and recorded every 2 h by the Incucyte device.

As for the wound healing assay, 1 × 10^5^ cells were seeded into a 12-well plate, and wounds were made by a scratcher (ibidi, WI, United States). The size of the wound was automatically captured every 2 h by the Incucyte ZOOM device and measured by ImageJ software. Each assay was repeated three times. Transwell assay was constructed with cell culture plates of 24 well 8.0 µm pore size (Falcon, CO, United States) with Matrigel (BD Biosciences, NJ, United States). 1x10^5^ cells were seeded into the upper chamber in 0.1 ml of serum-free RPMI-1640, and 0.5 ml of RPMI-1640 with 20% FBS was placed in the lower chamber as a chemoattractant. Invasion cells on the other side of the membrane were fixed and stained with crystal violet for 5 min after 48 h culture. Each assay was repeated three times.

### Cell Cycle Assays

Cell cycle assay was constructed with Cell Cycle Staining Solution (Multi Sciences, China) according to the manufacturer’s protocol. The flow cytometry results were measured by the Beckman device (BD Biosciences, NJ, United States) and analyzed with Flow jo 10.0 software. Each assay was repeated three times.

### Immunohistochemistry Assay

Each sample was deparaffinized for antigen retrieval using sodium citrate (pH 6.0) for 10 min and subsequent incubation with the respective primary antibody: RAB17 (Proteintech, #17501-1-AP), RAB34 (Affinity, #AF9174). Antibodies were used at 1:100 dilution. The reaction was developed using hematoxylin for counterstaining for 2 min. In all cases, sections from normal colonic mucosa distant from the tumor site were used as negative controls.

### Colony Formation Assays

For colony formation assay, cells were seeded in a six-well plate (500 cells per well) and allowed for growth for 2 weeks. The culture medium was refreshed every 5 days. At the end of the experiment, colonies were stained by crystal violet.

### Structure Similarity Analysis

Structure similarity of RABs were analyzed through sequence alignment. The sequence of each RABs were obtained from UniProt (https://www.uniprot.org/) and aligned by Clustal Omega online tool (Madeira et al., 2019). The phylogenetic relationships are obtained by the neighbor-joining program in the Jalview package. Finally, sequence conservation, consensus residues, and phylogenetic tree were visualized by Jalview (Waterhouse et al., 2009).

### Bioinformatic Analysis

GEPIA (http://gepia.cancer-pku.cn/index.html) was used to analyze RABs expression in normal and tumor tissues, as well as the correlation between RABs expression and patient prognosis. Genetic alterations of RABs were analyzed by the c-Bio Cancer Genomics Portal (https://www.cbioportal.org/) website, and the tab OncoPrint shows an overview of genetic changes for each sample in RABs. GeneMANIA (http://www.genemania.org) was used to predict the related genes of RABs. Metascape (https://metascape.org) was used to enrich the functional pathways of RABs. TIMER (https://cistrome.shinyapps.io/timer/) was used to evaluate the level of tumor-infiltrating immune cells (TIIC). The correlation between CXCL11 and TILs was measured by Spearman’s test. Selected RABs were input via the “Immune module” and “Exploration module.” Home for Researchers (https://www.home-for-researchers.com) was used to evaluate the correlation between RABs expression and tumor mutation burden (TMB). Gene Set Enrichment Analysis (GSEA) analysis was conducted with GSEA preranked tool (http://software.broadinstitute.org/GSEA/msigdb/annotate.jsp).

### Statistical Analysis

Statistical analysis was constructed using the R 3.6.3 version and GraphPad Prism 8.0.1. Statistical significance was analyzed by a two-tailed Student’s *t*-test. Spearman’s correlation was performed to analyze correlations. Kaplan-Meier Survival analysis was performed with a log-rank test. Univariate and multivariate survival analyses were performed using the Cox regression analyses model. Statistical significance was defined by a two-tailed *p* < .05.

## Results

### Gene Expression, Genetic Alteration, and Sequence Alignment of RABs in Colorectal Cancer

**FIGURE 1:**
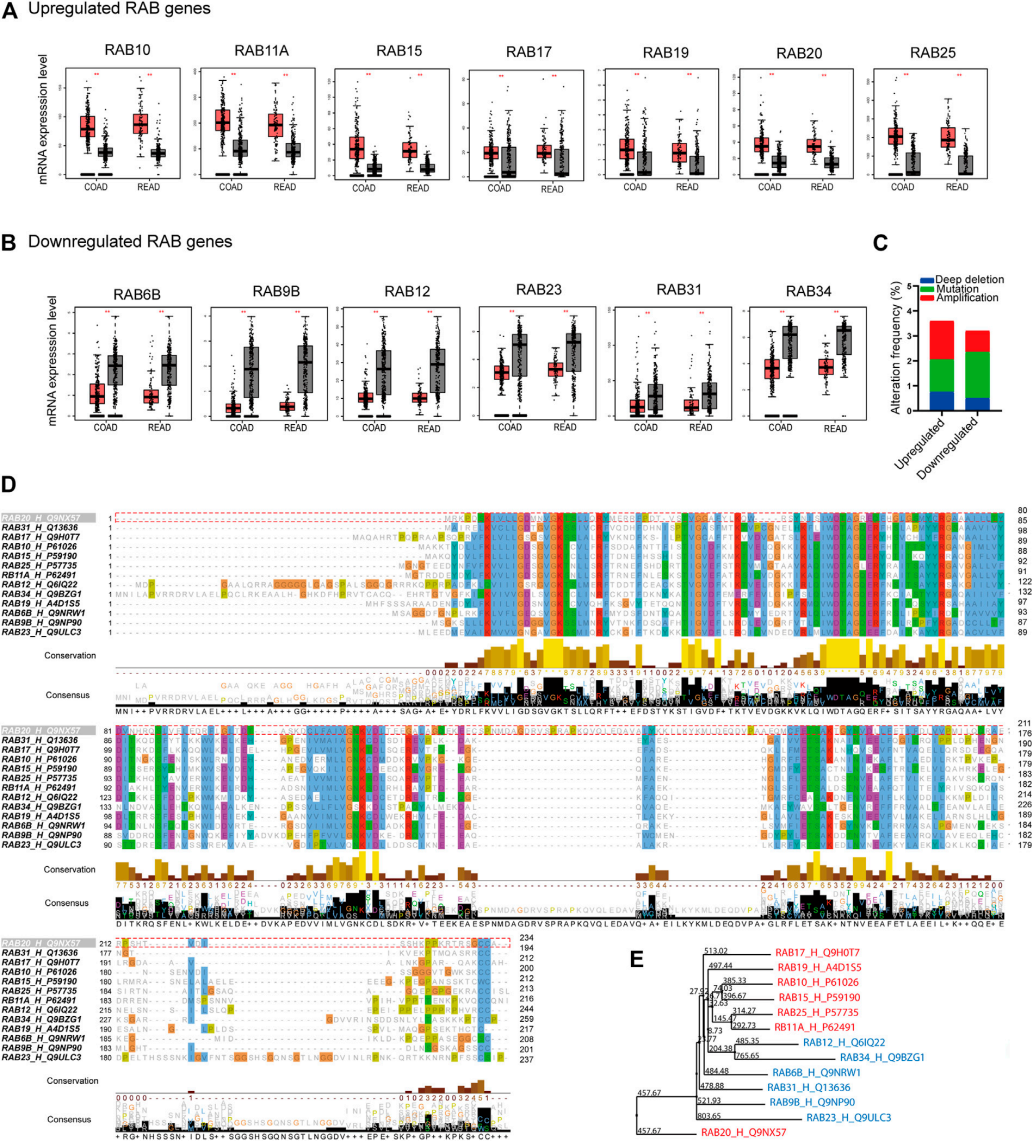
Differentially expressed RABs, genetic alteration, and sequence alignment of the RABs in colorectal cancer. **(A)** 7 RABs (RAB10, RAB11A, RAB15, RAB17, RAB19, RAB20, and RAB25) were significantly upregulated in tumor tissues. **(B)** 6 RABs (RAB6B, RAB9B, RAB12, RAB23, RAB31, and RAB34) were significantly downregulated in tumor tissues. Red and grey bar represents tumor and normal tissues, respectively. Tumor tissues (COAD: *n* = 275, READ: *n* = 92) and normal tissues (COAD: *n* = 349, READ: *n* = 318). **(C)** Genetic alterations frequency of each RABs in 2544 CRC patients. **(D)** Sequence alignment of the RABs. **(E)** Phylogenetic tree of the maximum likelihood analysis. ^**^ *p* < .01.

We first analyzed the mRNA expression profiles of 62 RABs in CRC using The Cancer Genome Atlas (TCGA) database and Genotype-Tissue Expression GTEx database through the GEPIA website portal. 13 RABs showed significantly different expressions in tumor tissues as compared to normal tissues in colon cancer (COAD) and rectal cancer (READ). Among them, seven were significantly upregulated, including RAB10, RAB11A, RAB15, RAB17, RAB19, RAB20, and RAB25 (Figure 1A; Supplementary Figure 1A), while the other six, RAB6B, RAB9B, RAB12, RAB23, RAB31, and RAB34, were significantly downregulated (Figure 1B; Supplementary Figure 1B). The correlation of the RABs with each other was analyzed via the R ggplot 2 (Supplementary Figure 1C).

Next, we examined the genetic alterations of RABs in 2544 CRC patients using the c-BioPortal tool. RAB20 possesses the highest alteration frequency (1.3%) which was mainly due to amplification alteration in 34 cases (Supplementary Figure 2B). Overall, the mutation and deep deletion alteration occupied a higher proportion in downregulated-RABs, which may contribute to their downregulation at transcriptional level (Figure 1C).

Sequence alignment was then performed to evaluate the structural similarity of RABs. The RABs exhibit moderate sequence identity (∼30–40%) and possess some highly conserved motif (Figure 1D; Supplementary Figure 2A). The downregulated RABs and the upregulated RABs formed relative distinct groups in phylogenetic analysis (Figure 1E). The phylogenetic distance between RAB20 and other RABs is the largest, which may be due to the highest genetic alteration frequency found in RAB20 (Supplementary Figure 2B). Previous studies have shown that the up-regulated RABs are usually located in endosome system (E), whereas, the down-regulated RAB genes are usually located in Golgi (G) (Langemeyer et al., 2018; Homma et al., 2021) (Supplementary Figure 2C). The above results indicated that though the RABs have a high degree of structural homology, the functions may vary depending on their subcellular localization.

### Correlation Between RABs Expression and Molecular Signatures, and GESA Enrichment Analysis

**FIGURE 2:**
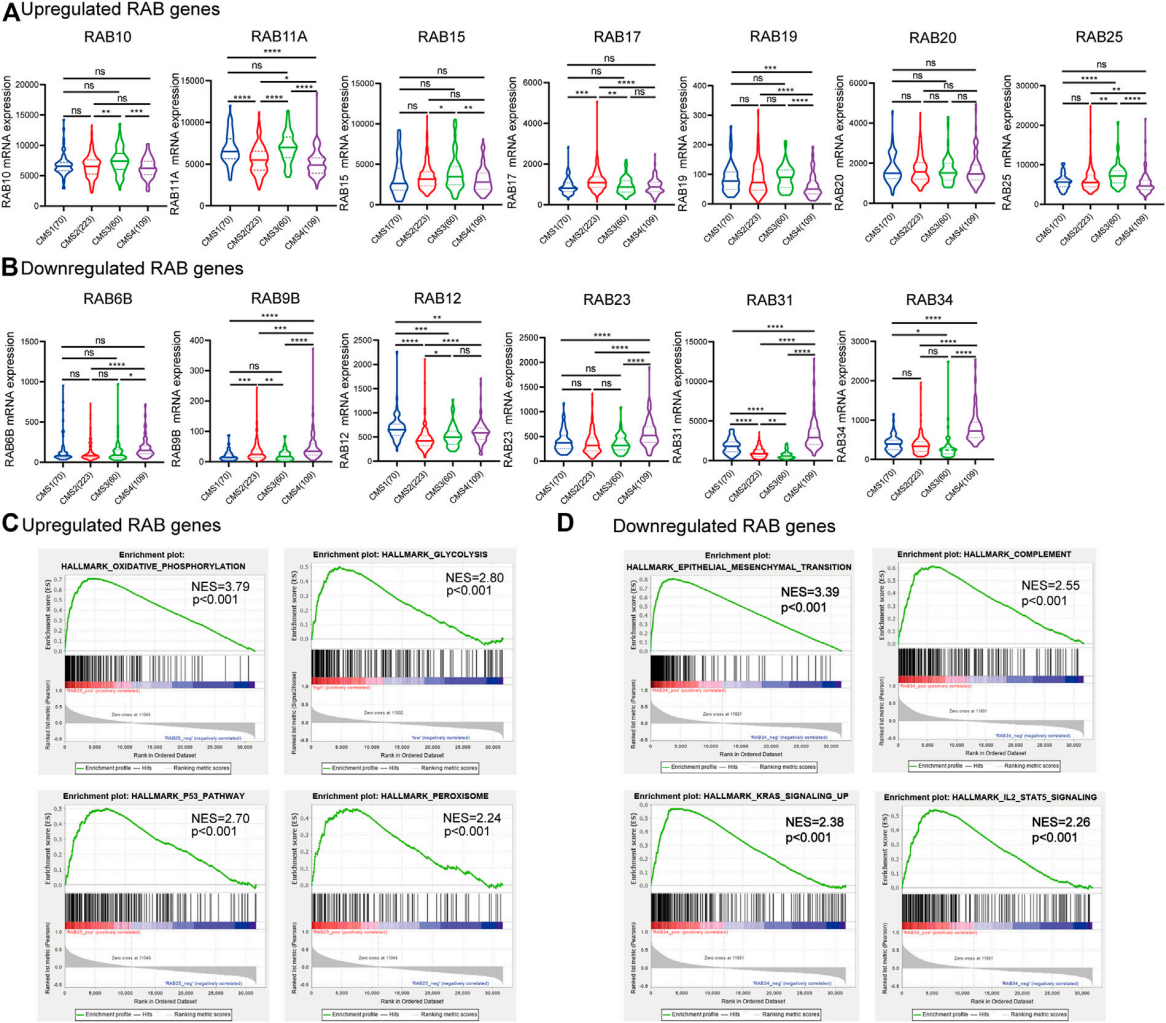
Correlation between RABs expression and CMSs and GESA enrichment analysis in CRC patients. **(A)** 7 RABs (RAB10, RAB11A, RAB15, RAB17, RAB19, RAB20, and RAB25) were significantly correlated with CMS3. **(B)** 6 RABs (RAB6B, RAB9B, RAB12, RAB23, RAB31, and RAB34) were significantly correlated with CMS4. Statistical significance was defined by a two-tailed *t*-test. **(C–D)** Gene set enrichment analysis of RABs. ^*^ *p* < .05, ^**^ *p* < .01, ^***^ *p* < .001, ^****^ *p* < .0001.

Transcriptional profiling has identified four consensus molecular subtypes (CMSs) of CRC possessing distinct molecular signatures and prognostic profiles (Guinney et al., 2015). CMS1 is characterized with microsatellite unstable and strong immune activation; CMS2 is characterized with WNT and MYC signaling activation; CMS3 shows evident metabolic dysregulation; and CMS4 is associated with TGFβ activation, stromal invasion, and angiogenesis. Therefore, we investigated the correlations between RABs expression and CMSs. We found that a high level of the upregulated-RABs was significantly associated with the metabolic CMS3 subtype (Figure 2A), implying their involvement in the epithelial and metabolic dysregulation. Interestingly, the downregulated-RABs were significantly highly expressed in mesenchymal CMS4 tumors (Figure 2B), which are usually diagnosed at more advanced stages and more aggressive and metastatic than other CMS subtypes. The results indicated that though downregulated-RABs are low expressed in tumor tissues compared to normal tissues, their upregulation might result in a more aggressive cancer progression.

Consistently, GESA analysis showed that the upregulated-RABs mainly were enriched with metabolic-related pathways, while, the downregulated-RABs were enriched with EMT and immune-related pathways (Figures 2C,D).

**FIGURE 3:**
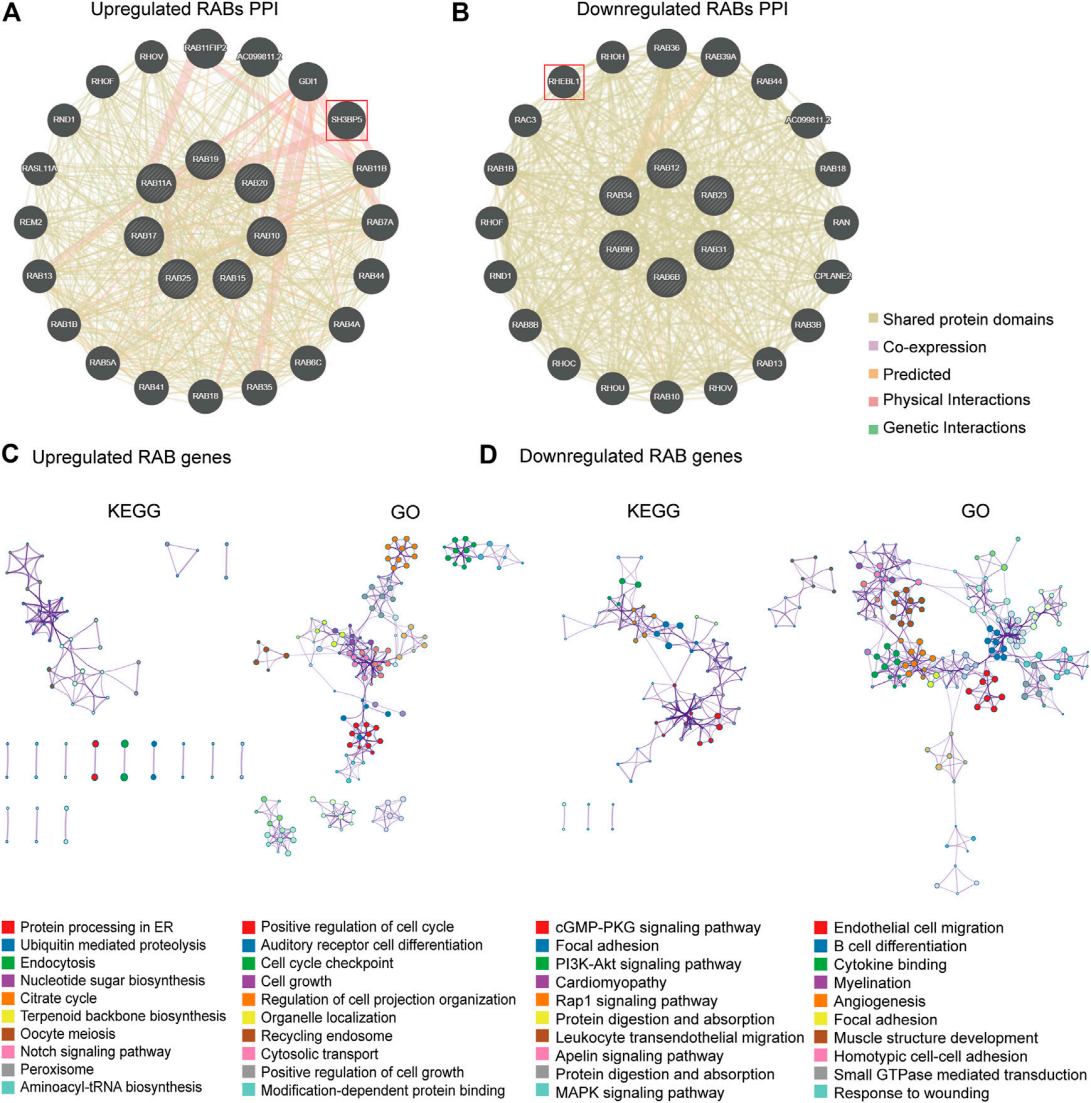
PPI, KEGG, and GO enrichment analysis for RABs. **(A–B)** The protein interaction network of RABs related genes in CRC. **(C–D)** Top 10 enrichment terms in KEGG pathways and GO in RABs (*p*-value of each pathway were shown in the Supplementary Tables S1–4).

To further figure out the critical intracellular signaling pathways related to RABs in CRC, we then performed Protein interaction network (PPI), Gene Ontology (GO), and Kyoto Encyclopedia of Genes and Genomes (KEGG) pathway analysis. The PPI results showed that the SH3BP5 and RHEBL1 were most closely associated with upregulated-RABs and downregulated-RABs, respectively (Figures 3A,B). SH3BP5 sustained the JNK activity and played a critical role in modulating cell death and cell cycle (Win et al., 2018; Lucero et al., 2019). RHEBL1 activated NFkB-mediated gene transcription and play an important role in the immune process (Yuan et al., 2005). The top 100 genes that were most positively relevant in the RABs were used for KEGG and GO enrichment analyses. Consistent with the PPI analysis results, cell cycle-related pathways were significantly enriched by upregulated-RABs (Figure 3C), whereas, immune-related pathways were enriched by downregulated-RABs (Figure 3D).

### Correlation Between RABs Expression and Immunotherapy Biomarkers

**FIGURE 4:**
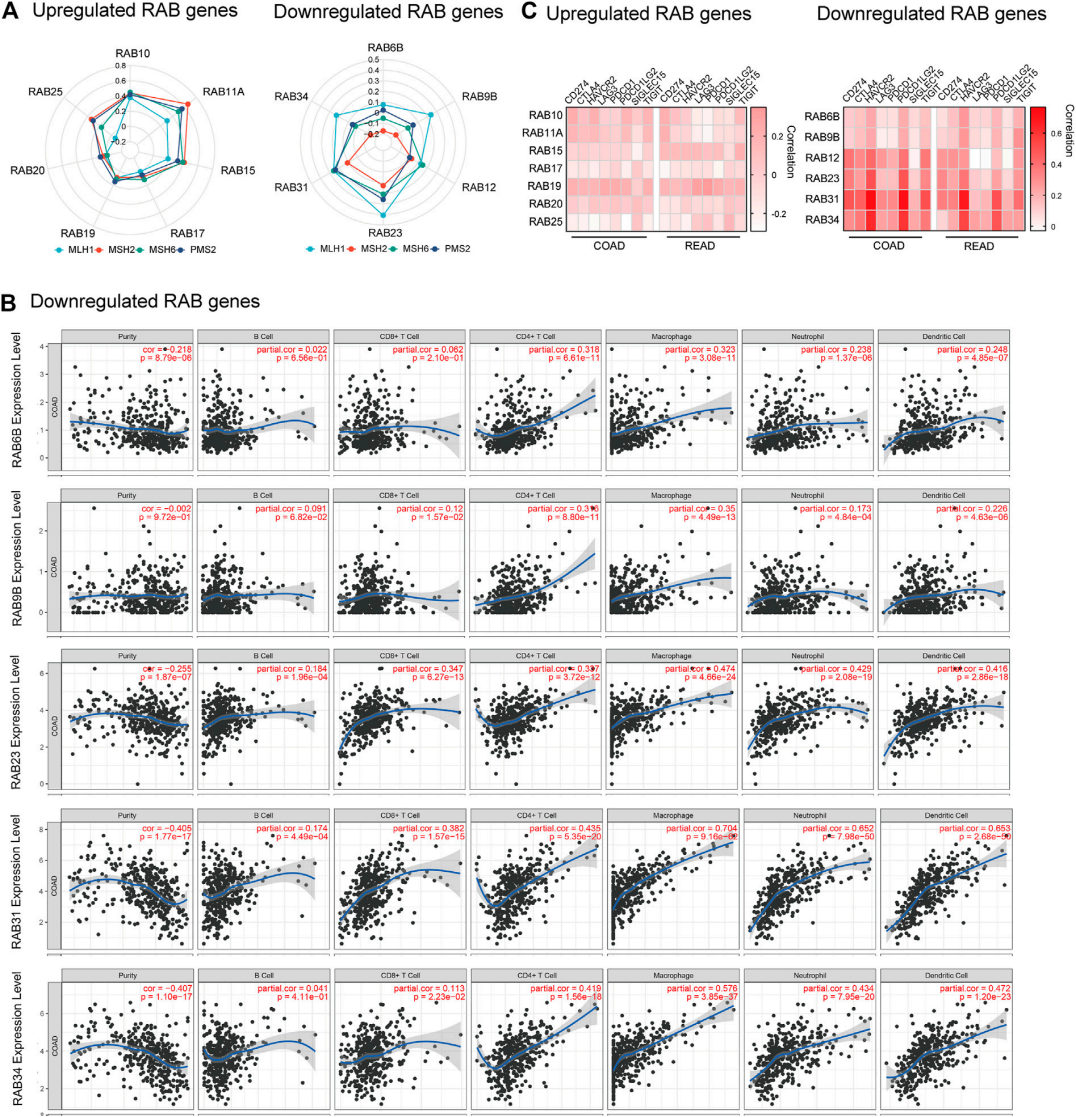
Correlation between RABs expression and immunotherapy biomarkers in CRC patients. **(A)** Correlation between the expression of RABs and MMR genes (*p*-value of each pathway were shown in Supplementary Tables S5–6). **(B)** Correlation between RABs and immune cells infiltration levels in COAD. **(C)** Correlation between the expression of RABs and immune checkpoint genes. Spearman’s correlation coefficients were shown above the bar graphs.

Immunotherapy has shown impressive results in patients with mismatch repair deficient (dMMR) or microsatellite instability-high (MSI-H) CRC (Supek and Lehner, 2015). Tumors that have a dMMR–MSI-H signature are usually associated with a high tumor mutation burden (TMB) and immune cell infiltration (Mandal et al., 2019). Therefore, we firstly investigated the correlation between the expression of RABs and four MMR genes (MLH1, MSH2, MSH6, and PMS2) by the R ggradar and ggplot2. The results showed that the level of upregulated-RABs was positively correlated with the MMR genes in CRC. However, this correlation is not obvious in the downregulated-RABs (Figure 4A). Interestingly, we found that only the expression of downregulated-RABs was positively correlated with infiltrating levels of CD8^+^ T cells, CD4^+^ T cells, macrophage, neutrophils, and dendritic cells in CRC (Figure 4B; Supplementary Figure S3–4). Moreover, the overall correlation between RABs expression and TMB was not significant (Supplementary Figure S5). MMR is an intracellular process contributing to the fidelity of DNA synthesis and replication and is required for cell cycle regulation (Li et al., 2016). The positive correlation between MMR protein expression and upregulated-RABs may explain the above results that the cell cycle-related pathways were enriched in upregulated-RABs.

To further elucidate the possible role of RABs in regulating immunotherapy response, we then evaluated the correlation between RABs expression and immune checkpoint genes. We found that the expression of downregulated-RABs was more significantly positive correlated with immune checkpoint genes the R ggplot2 (Figure 4C), which is in line with the above analysis that immune-related pathways were enriched by downregulated-RABs.

### Correlation Between RABs Expression and Clinical Outcomes

**FIGURE 5:**
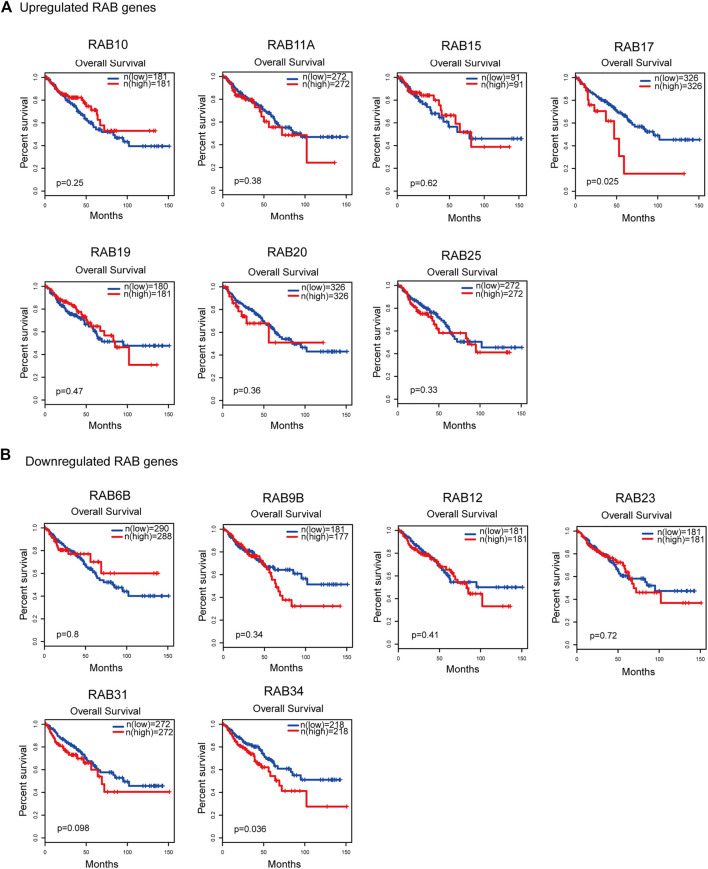
Kaplan–Meier plots for the overall survival of CRC patients stratified by the mRNA expression level of RABs. **(A)** The overall survival curves of the upregulated-RABs (RAB10, RAB11A, RAB15, RAB17, RAB19, RAB20, and RAB25). **(B)** The overall survival curves of the downregulated-RABs (RAB6B, RAB9B, RAB12, RAB23, RAB31, and RAB34).

**FIGURE 6:**
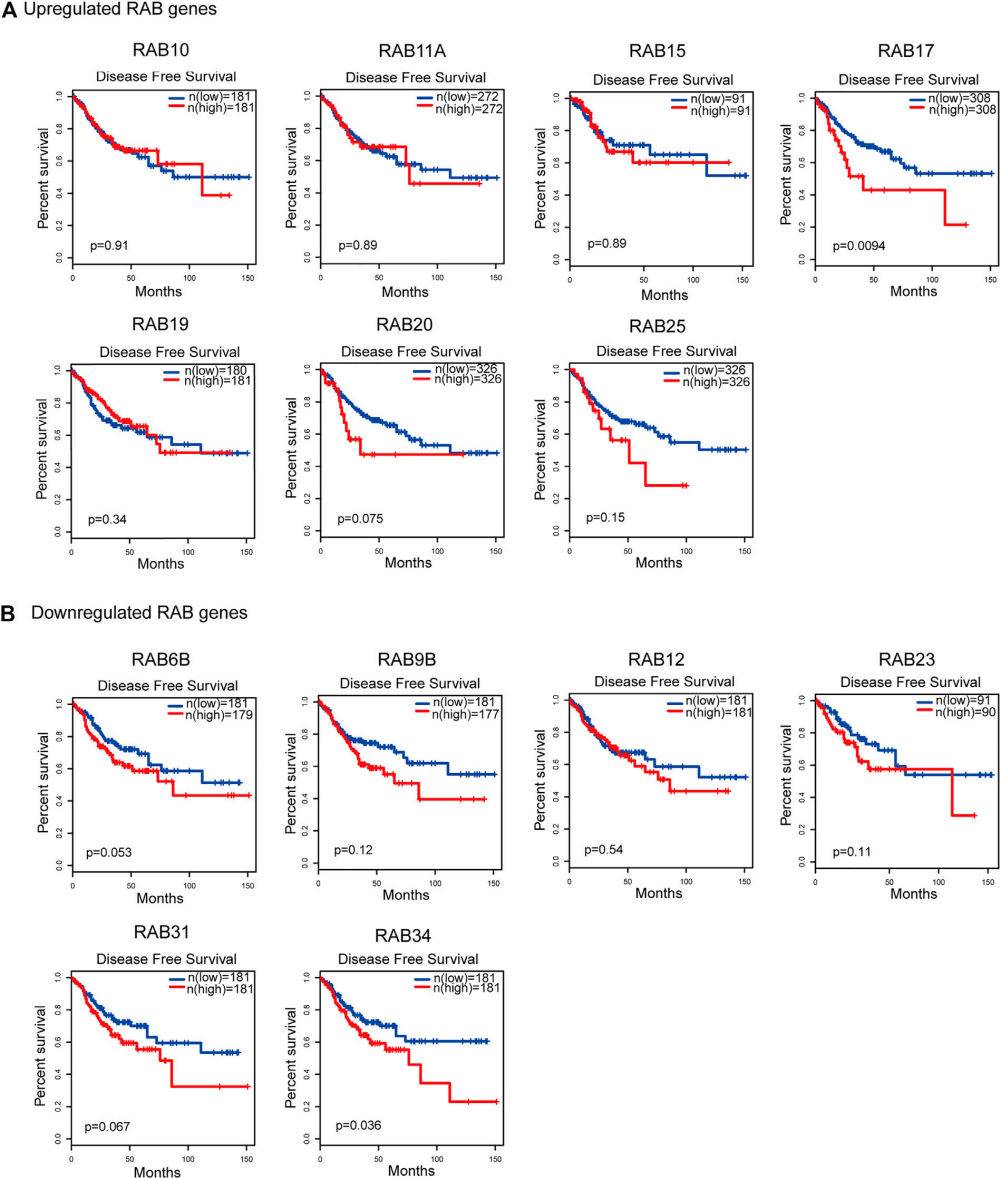
Kaplan–Meier plots for the disease-free survival of CRC patients stratified by the mRNA expression level of RABs. **(A)** The disease-free survival curves of the upregulated-RABs (RAB10, RAB11A, RAB15, RAB17, RAB19, RAB20, and RAB25). **(B)** The disease-free survival curves of the downregulated-RABs (RAB6B, RAB9B, RAB12, RAB23, RAB31, and RAB34).

To evaluate the prognostic value of differentially expressed RABs in CRC, we first analyzed the correlations between different RABs and clinical outcomes using the datasets from the GEPIA database. Except for RAB17 and RAB34, the expression of all the other RABs was not significantly associated with overall survival (OS) and disease-free survival (DFS). Kaplan–Meier survival curve showed that high mRNA levels of RAB17 and RAB34 were significantly associated with poor OS and DFS (Figures 5, 6). Thus, we further assessed the correlation between RAB17 and RAB34 mRNA expression and the clinicopathological features of TCGA CRC patients (*n* = 454). RAB17 was significantly associated with pN status, pM status, and TNM stage (Supplementary Table S7). RAB34 was significantly associated with pN status (Supplementary Table 8). Univariate and multivariate Cox regression analyses demonstrated that RAB17 was an independent prognostic factor for OS and DFS (Table 1; Supplementary Table S9), and RAB34 was an independent predictor for DFS (Table 2; Supplementary Table 10).

**TABLE 1:** Multivariate Cox regression analysis of RAB17 mRNA expression in CRC patients from TCGA.

Variable	OS		DFS	
	HR (95% CI)	*p* value	HR (95% CI)	*p* value
RAB17 expression (high vs. low)	1.792 (1.062–2.816)	0.028*	1.699 (1.016–2.840)	0.043*
Median age (<=68				
years vs . >68 years)	0.426 (0.255–0.711)	0.001**		
pN stage (N2 vs. N0+N1)	1.866 (1.086–3.205)	0.024*		
pM stage (M1 vs. M0)	4.109 (2.389–7.066)	0.001***	3.185 (1.724–5.885)	0.001***
Clinical stage (I+II vs.				
III+IV)				

* *p* < .05, **p < .01, ***p < .001.

**TABLE 2:** Multivariate Cox regression analysis of RAB34 mRNA expression in CRC patients from TCGA.

	OS		DFS	
Variable	HR (95% CI)	*p* value	HR (95% CI)	*p* value
RAB34 expression				
(high vs. low)			1.944 (1.170–3.231)	0.010*
Median age (<=68				
years vs . >68 years)	0.468 (0.282–0.777)	0.003**		
pT stage (T3–T4 vs. T1–T2)				
pN stage (N2 vs. N0+N1)				
pM stage (M1 vs. M0)	3.458 (1.869–6.398)	0.001***	3.419 (1.923–6.078)	0.001***
Clinical stage (I+II vs.	0.487 (0.264–0.900)	0.022*		
III+IV)				

* *p* < .05, **p < .01, ***p < .001.

### RAB17 Promotes Cell Proliferation via Cell Cycle Regulation in CRC Cells

**FIGURE 7:**
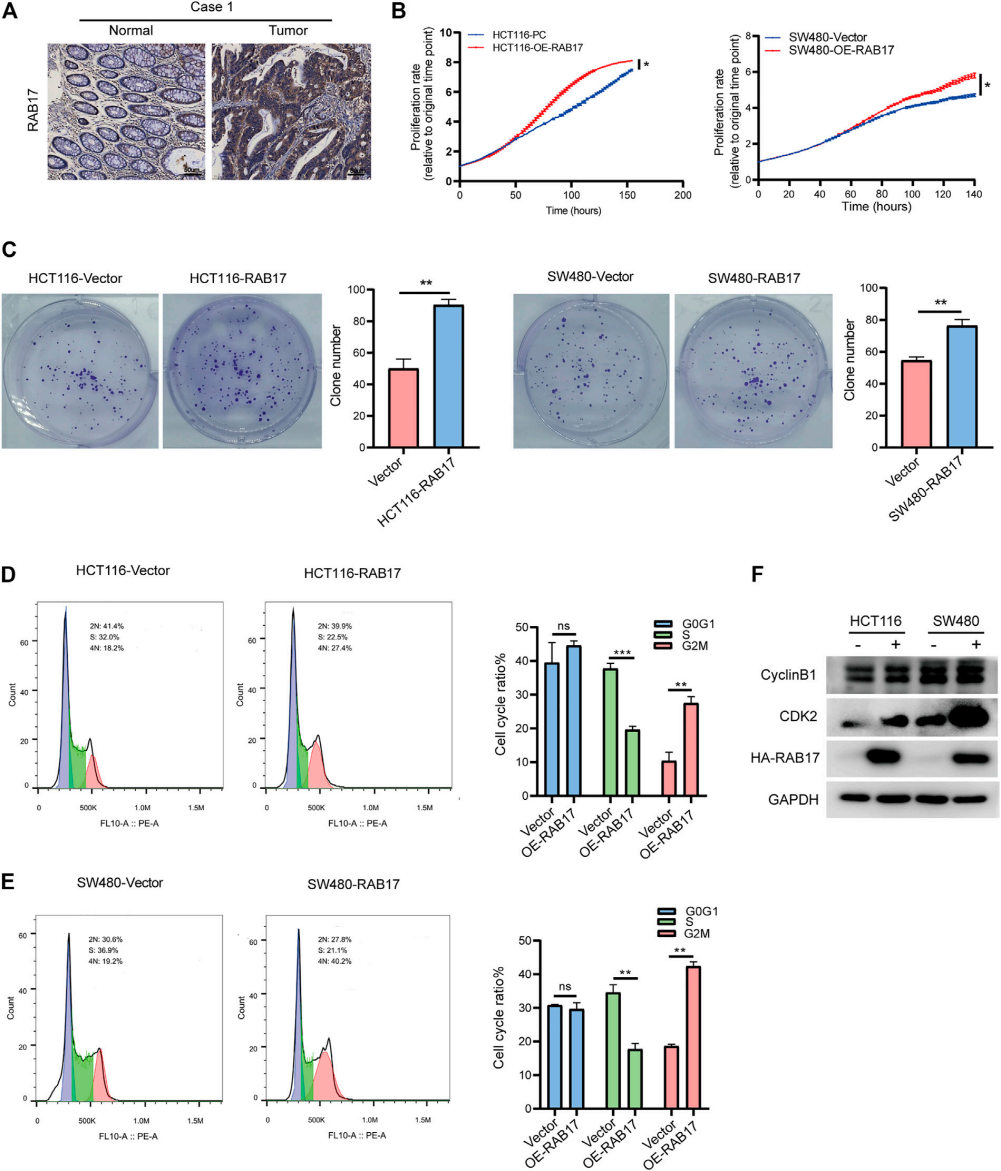
Overexpression of RAB17 promotes cell proliferation of CRC. **(A)** Immunohistochemical staining of patients with colorectal cancer. **(B)** Incucyte assay revealed cell proliferation in HCT116 cells and SW480 cells. **(C)** Overexpression of RAB17 analyzed the colony-forming ability of HCT116 cells and SW480 cells. **(D–E)** Overexpression of RAB17 analyzed cell population both in HCT116 cells **(D)** and SW480 cells **(E)** by flow cytometer. **(F)** Overexpression of RAB17 analyzed the protein levels of CDK2 and cyclin B1 in the HCT16 cells and SW480 cells. ^*^ *p* < .05, ^**^ *p* < .01, ^***^ *p* < .001.

We then investigated the role of RAB17 *in vitro.* Immunohistochemical staining showed that RAB17 was highly expressed in tumors (Figure 7A; Supplementary Figure S6A). Overexpression of RAB17 in HCT116 and SW480 cells significantly promoted cell growth (Figure 7B). Similarly, colony formation assay showed that overexpression of RAB17 in HCT116 and SW480 cells increased the colony number compared with the corresponding controls (Figure 7C). Our previous analysis showed that upregulated-RABs were highly correlated with the cell cycle, therefore, we investigated the involvement of RAB17 in regulating cell cycle progression using flow cytometry. The results showed that overexpression of RAB17 resulted in an increased percentage of cells in the G2M phase and a decreased percentage of cells in the S phase in HCT116 and SW480 cells (Figures 7D,E). Overexpression of RAB17 significantly increased the protein levels of CDK2 and cyclinB1, which are the cell cycle checkpoint genes regulating S to G2M transition, in the HCT16 cells and SW480 cells (Figure 7F). These results demonstrated that RAB17 could promote CRC cells proliferation via regulating cell cycle progression.

### RAB34 Overexpression Promotes Cell Migration and Invasion

**FIGURE 8:**
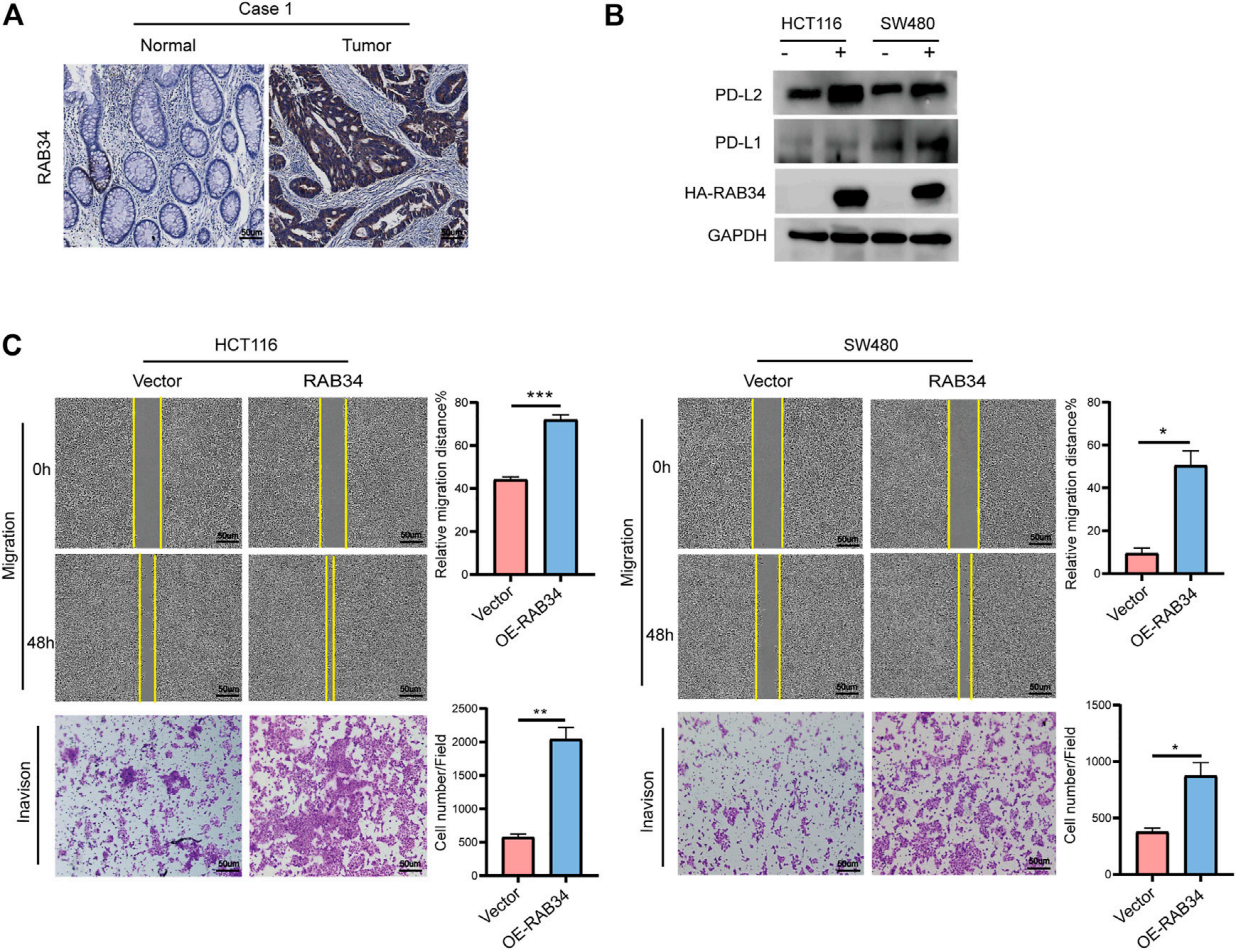
Overexpression of RAB34 increased PD-L1 and PD-L2 expression of CRC. **(A)** Immunohistochemical staining of patients with colorectal cancer. **(B)** Overexpression of RAB34 analyzed the protein levels of PDL-1 and PD-L2 in the HCT16 cells and SW480 cells. **(C)** Overexpression of RAB34 analyzed the migration and invasion in the HCT16 cells and SW480 cells. ^*^ *p* < .05, ^**^ *p* < .01, ^***^ *p* < .001.

Our immunohistochemistry analysis showed strong staining signals of RAB34 in CRC tumor tissues but low in normal tissues (Figure 8A; Supplementary Figure S6B). Given that RAB34 was significantly upregulated in CMS4 tumors, which showed mesenchymal activation and high invasion ability, we assessed if RAB34 overexpression in CRC cells can promote cell migration and invasion. The wound-healing and transwell assay results showed that overexpression of RAB34 remarkably promotes cell migration and invasion in HCT116 and SW480 cells (Figure 8C). As shown in Figure 4C, the expression of RAB34 was positively correlated with immune checkpoint genes and RAB34, we chose PD-L1 and PD-L2 for further verification by western blot in CRC cells. The results showed that RAB34 overexpression can significantly increase the protein levels of PD-L1 and PD-L2 in CRC cells (Figure 8B). These results combined with the survival analysis indicated that RAB34 is an unfavorable prognostic factor, more importantly, targeting RAB34 might provide a novel therapeutic strategy to improve the responses to anti-PD-1 therapy in CRC.

## Discussion

More than 1.2 million patients are diagnosed with colorectal cancer every year and about 600,000 die of the disease (Brenner et al., 2014). Approximately 35–45% of CRC patients are RAS mutant, which is associated with therapy resistance (Wong et al., 2020). It is important to better explore the function of the RAS family proteins and the related pathways driven by activated RAS, therefore, to find new drugable targets for clinical treatment of CRC.

Rab proteins, which belongs to the RAS family, are vital components of the membrane trafficking system that controls secretion, transport, recycling, and degradation of many tumor-associated proteins, such as beta-integrins, epidermal growth factor receptor (EGFR), and matrix metalloproteinases (MMPs) (Dozynkiewicz et al., 2012; Anand et al., 2020). The role of RABs in the shedding of extracellular vesicles has also been reported (Peinado et al., 2012; Recchi and Seabra, 2012). Aberrant expression of RABs have been found in various cancers (Wang et al., 2020), but the roles of RABs in colorectal cancer are less well understood.

In this study, we evaluate the role of RABs in CRC using public datasets and verify their functions *in vitro*. We found that though the RABs exhibit a high degree of sequence similarity, but the function are vary may be due to their different subcellular location. The RABs play critical roles in regulating cell cycle progression, immune cell infiltration, and might be predictive markers of immunotherapy and patient survival in CRC.

According to the expression level of RABs in tumors compared with that in normal tissues, we divided them into the upregulated and downregulated groups. We found that the upregulated-RABs identified in CRC (RAB10, RAB11A, RAB15, RAB17, RAB19, RAB20, and RAB25) were significantly associated with the metabolic CMS3 subtype and cell cycle regulation. The CMS3 tumors often display remarkable metabolic deregulation with higher KRAS mutations (68%) (Lal et al., 2018; Smeby et al., 2018), which is consistent with the GSEA enrichment analysis. Metabolic plasticity is critical for DNA damage repair and cell cycle regulation, which subsequently affect cancer cell survival, growth, and proliferation (Alcalá et al., 2020; Kumarasamy et al., 2021). Previous studies have shown that RAB10, RAB11A, RAB17, and RAB25 could promote cell proliferation in different cancers (Li et al., 2015; Du J. et al., 2020; Zhang et al., 2020). Elevated expression of Rab25 was correlated with poor prognosis and aggressiveness of renal, lung, breast, ovarian, and other cancers. However, the tumor suppressor function of Rab25 was reported in several cancers, such as colorectal cancer, indicating the tumor type-specific function of Rab25 (Hong et al., 2018; Jeong et al., 2018; Cho and Lee, 2019; Temel et al., 2020). Our results reveal that RAB17-overexpressed CRC cells exhibited an increase in the percentage of cells in the G2/M phase. Furthermore, RAB17 overexpression induced an increase of cyclin B and CDK2 protein levels, which are important checkpoint genes in controlling the S to G2/M transition. More importantly, we found that RAB17 is an independent marker significantly associated with OS and DFS in CRC. It is believed that cytotoxic cancer chemotherapy drugs usually kill dividing cells that proliferate fast (He et al., 2019). Collectively, these observations suggested that the upregulated RABs, especially RAB17, could regulate cell cycle progression and might be useful prognostic markers to be used to stratify patients into a group that would benefit from chemotherapy treatment.

As to the downregulated-RABs (RAB6B, RAB9B, RAB12, RAB23, RAB31, and RAB34), we found their expression levels were significantly correlated with immune cells infiltration. Consistently, the GESA analysis showed that the downregulated-RABs are enriched with immune-related pathways including IL2-STAT5 signaling and complement related pathways. Studies have shown that RAB23 and RAB31 participate in the autophagy process, which play a significant role in immunity (Zheng et al., 2017). RAB34 is associated with lysosomal distribution, hence affecting antigen presentation by dendritic cells and CD8^+^ T activation (Alloatti et al., 2015). Moreover, the expression levels of immune checkpoint genes are key factors affecting immunotherapy response. Our analysis revealed that the level of downregulated RABs are positively correlated with immune checkpoint genes expression. Furthermore, *in vitro* verification showed that RAB34-overexpression induced a significant upregulation of PD-L1 and PD-L2, the two ligands of PD-1. Therefore, we speculated that the downregulated-RABs are not only be involved in regulating the transportation and membrane translocation of the immune checkpoint genes but may also modulate the tumor microenvironment by recruiting immune cells to the tumor site. The level of down-regulated RABs may help to predict the immunotherapy response.

Among the 13 differentially expressed RABs, RAB17 and RAB34 were independent markers significantly associated with poor OS and DFS. Interestingly, though the mRNA level of RAB34 was downregulated in tumor tissues, a high level of RAB34 was significantly associated with poor OS and DFS. This could have different explanations. First, we found the downregulated RABs are highly expressed in CMS4 CRC, which are usually diagnosed at the late clinical stage and have the worst survival, suggesting that their upregulation is associated with a more aggressive phenotype. Second, there may be unknown post-transcription modification affecting the expression and functions of these RABs. Supporting this notion, though RAB3A mRNA could be detected, a lack of RAB3A immunostaining in pancreatic ductal adenocarcinoma (PDAC) tissues were reported (Dragomir and Calin, 2018). It is also likely that other interacting proteins of these RABs may also be aberrantly expressed in CRC and have an impact on their functions. Specific experiments need to be carried out in further research.

In summary, our study provides initial data elucidating the possible role of RABs in CRC. RABs might be important markers in predicting immunotherapy response and patient survival. Further studies using clinical samples and different models should be considered to explore their utility and underlying mechanisms as therapeutic targets and clinical biomarkers.

## Data Availability

The datasets presented in this study can be found in online repositories. The names of the repository/repositories and accession number(s) can be found in the article/Supplementary Material.

## Abbreviations

AP, autophagosome; C, cilium; COAD, colon cancer; CRC, colorectal cancer; CMS, consensus molecular subtypes; DCs, dendritic cells; DFS, disease-free survival; dMMR, mismatch repair deficient; E, endosome; EE, early endosome; ER, endocytic recycling; EGFR, epidermal growth factor receptor; G, golgi; GO, gene ontology; GTPases, small guanosine triphosphatases; KEGG, Kyoto encyclopedia of genes and genomes; LE, late endosome; LY, lysosome; MS, melanosome; MMPs, matrix metalloproteinases; MMR, mismatch repair; MSI-H, microsatellite instability-high; OS, overall survival; P, peroxisome; PM, plasma membrane; PS, phagosome; PPI, protein interaction network; RABs, RAB proteins; READ, rectal cancer; RE, recycling endosome; TE, tubular endosome. TGN, *trans*-golgi network; TCGA, The cancer genome atlas; TMB, tumor mutation burden.
