# Supplementary material for: The Role of RAB GTPases and Its Potential in Predicting Immunotherapy Response and Prognosis in Colorectal Cancer
Source: Front Genet. 2022 Jan 28;13:828373. doi: 10.3389/fgene.2022.828373 (PMC8833848; doi:10.3389/fgene.2022.828373)
Supplement: Supplementary file 1 [file DataSheet1.ZIP › Supplementary Figures/Supplementary Figure 1. mRNA expression analysis of RABs in CRC samples through the GEPIA and the Spearmaní»s correction..docx]

**Supplementary Figure 1.** mRNA expression analysis of RABs in CRC samples through the GEPIA and the Spearman’s correction. Based on the significant (P< 0.05) alteration of RABs in COAD (T=275, N=349) and READ (T=92, N=318), the RABs were divided into two subsets: **(A)** Upregulated-RABs (RAB10, RAB11A, RAB15, RAB17, RAB19, RAB20, and RAB25). **(B)** Downregulated-RABs (RAB6B, RAB9B, RAB12, RAB23, RAB31, and RAB34). **(C)** The correlation of RAB genes with each other.

**
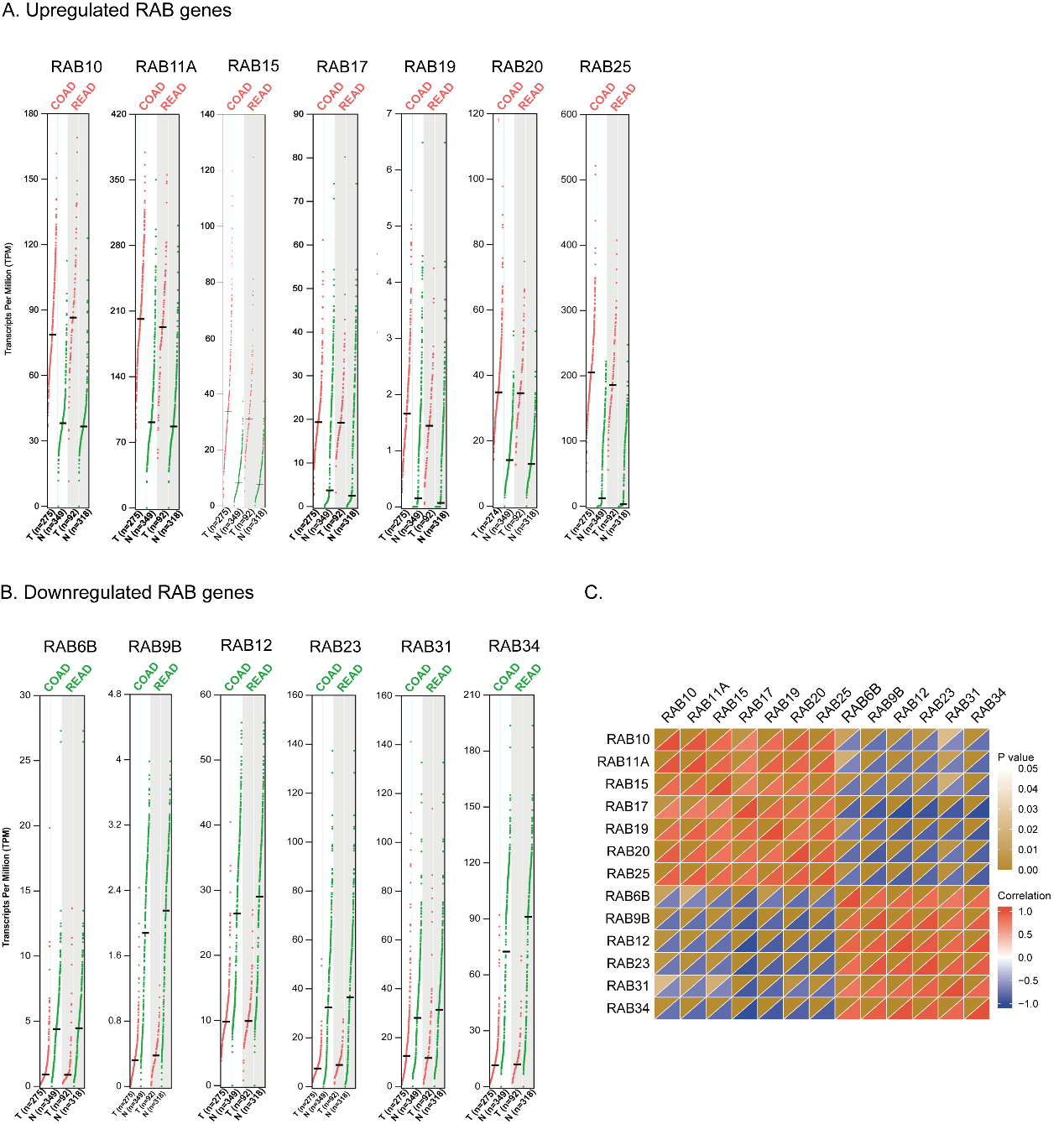
**
