# Supplementary material for: The Role of RAB GTPases and Its Potential in Predicting Immunotherapy Response and Prognosis in Colorectal Cancer
Source: Front Genet. 2022 Jan 28;13:828373. doi: 10.3389/fgene.2022.828373 (PMC8833848; doi:10.3389/fgene.2022.828373)

**Supplementary Figure 2.** The structural similarity through sequence alignment, genetic alteration, and subcellular localization of RABs. (A) The sequence similarity of RABs. (B) Genetic alterations frequency of each RABs from 2544 CRC patients. (C) The subcellular localization of RABs. AP, autophagosome; E, endosome; G, Golgi; LY, lysosome; MS, melanosome; P, peroxisome; PM, plasma membrane; PS, phagosome; SG, secretory granule; TGN, trans-Golgi network.


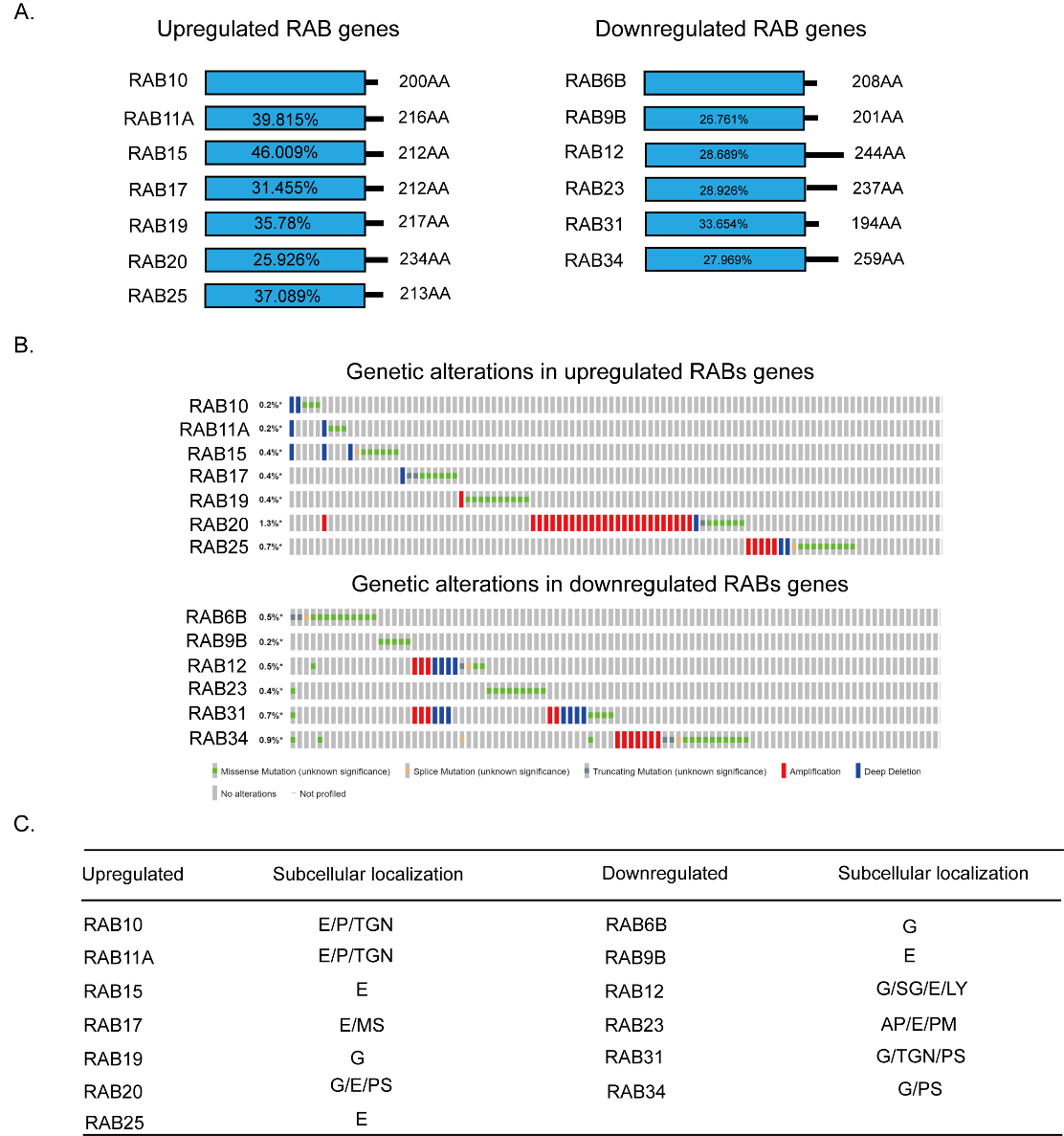

Supplement: Supplementary file 1 [file DataSheet1.ZIP › Supplementary Figures/Supplementary Figure 2. The structural similarity through sequence alignment, genetic alteration, and subcellular localization of RABs. .docx]
