# Supplementary material for: The Role of RAB GTPases and Its Potential in Predicting Immunotherapy Response and Prognosis in Colorectal Cancer
Source: Front Genet. 2022 Jan 28;13:828373. doi: 10.3389/fgene.2022.828373 (PMC8833848; doi:10.3389/fgene.2022.828373)
Supplement: Supplementary file 1 [file DataSheet1.ZIP › Supplementary Figures/Supplementary Figure 3. Correlation between upregulated-RABs expression and immune cells infiltration level in COAD and READ..docx]

**Supplementary Figure 3.** Correlation between upregulated-RABs expression and immune cells infiltration level in COAD and READ. Upregulated-RABs expression correlates with immune cells infiltration levels of B cells, CD8+T cells, CD4+T cells, macrophage, neutrophils, and dendritic cells.

**
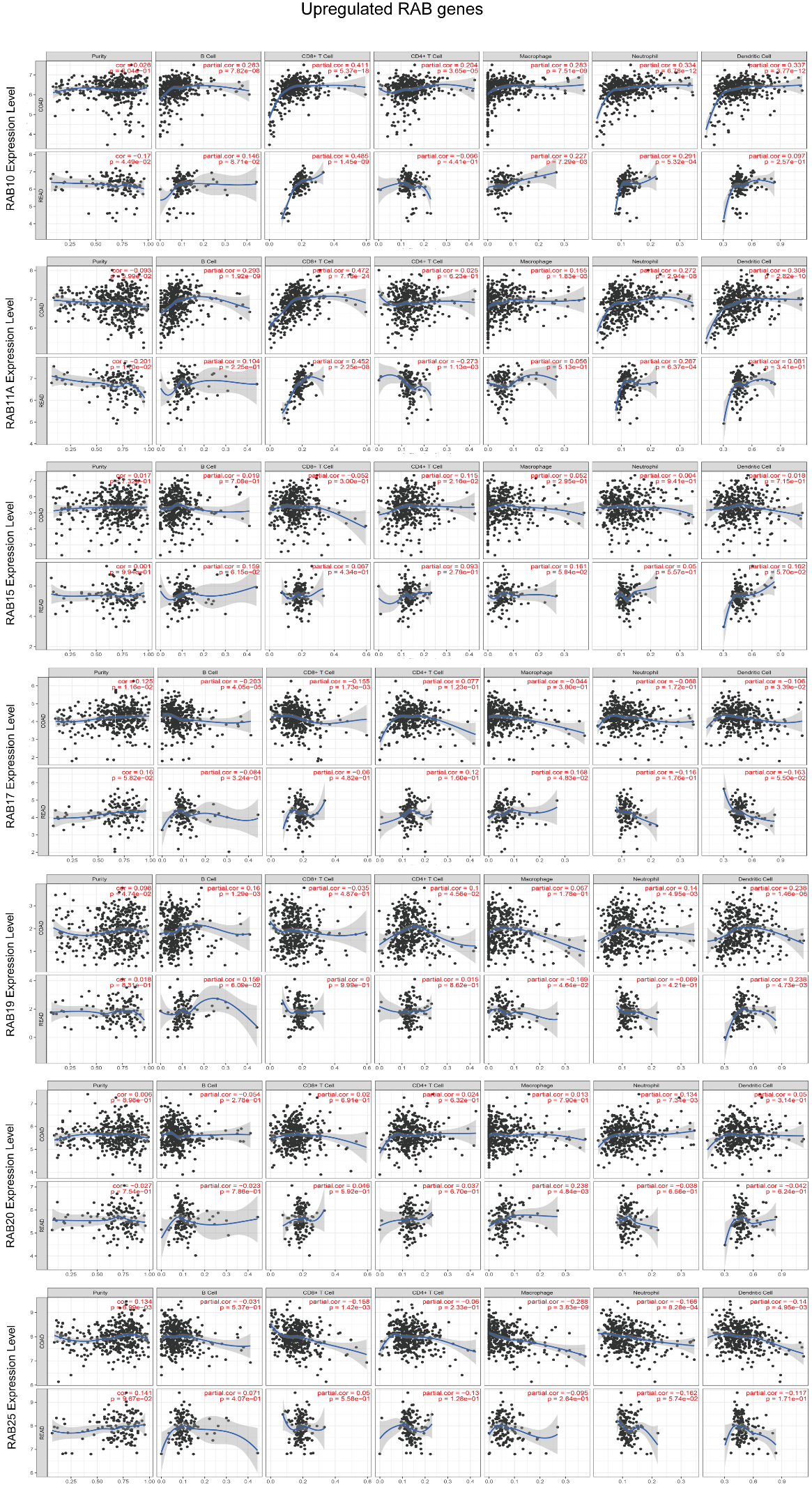
**
