# Supplementary material for: The Role of RAB GTPases and Its Potential in Predicting Immunotherapy Response and Prognosis in Colorectal Cancer
Source: Front Genet. 2022 Jan 28;13:828373. doi: 10.3389/fgene.2022.828373 (PMC8833848; doi:10.3389/fgene.2022.828373)
Supplement: Supplementary file 1 [file DataSheet1.ZIP › Supplementary Figures/Supplementary Figure 5. Correlation analysis of RABs expression and TMB..docx]

**Supplementary Figure 6.** Correlation analysis of RABs expression and TMB. The horizontal axis in the figure represents the expression distribution of the gene, and the ordinate is the expression distribution of the TMB score. The density curve on the right represents the distribution trend of the TMB score; the upper-density curve represents the distribution trend of the gene. a. Upregulated-RABs. b. Downregulated-RABs.

**
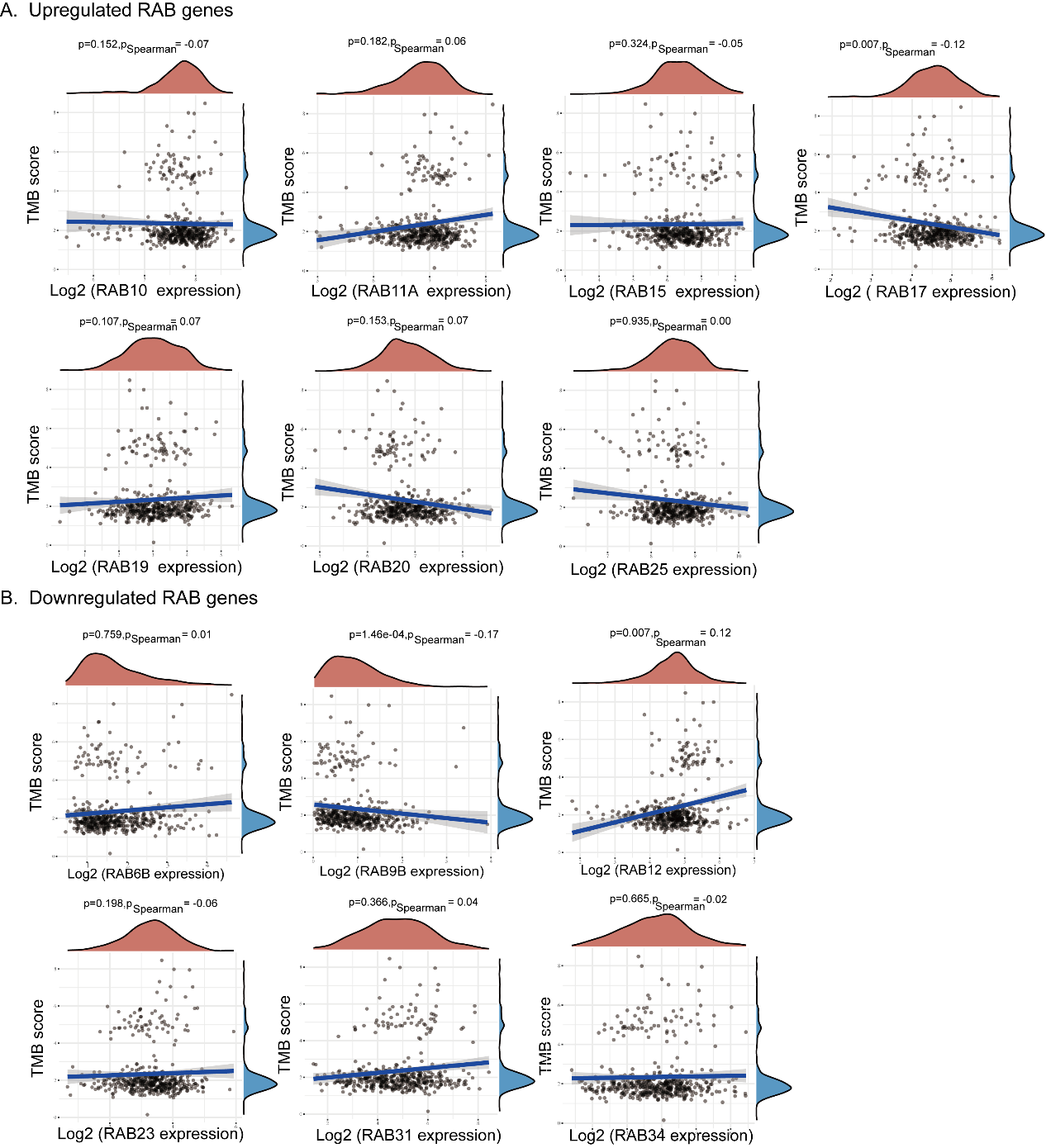
**
