# Supplementary material for: The Role of RAB GTPases and Its Potential in Predicting Immunotherapy Response and Prognosis in Colorectal Cancer
Source: Front Genet. 2022 Jan 28;13:828373. doi: 10.3389/fgene.2022.828373 (PMC8833848; doi:10.3389/fgene.2022.828373)
Supplement: Supplementary file 1 [file DataSheet1.ZIP › Supplementary Figures/Supplementary Figure 6. Immunohistochemical staining of patients with colorectal cancer in RAB17 and RAB34.docx]

**Supplementary Figure 7.** Immunohistochemical staining of patients with colorectal cancer in RAB17 (A) and RAB34 (B).

**
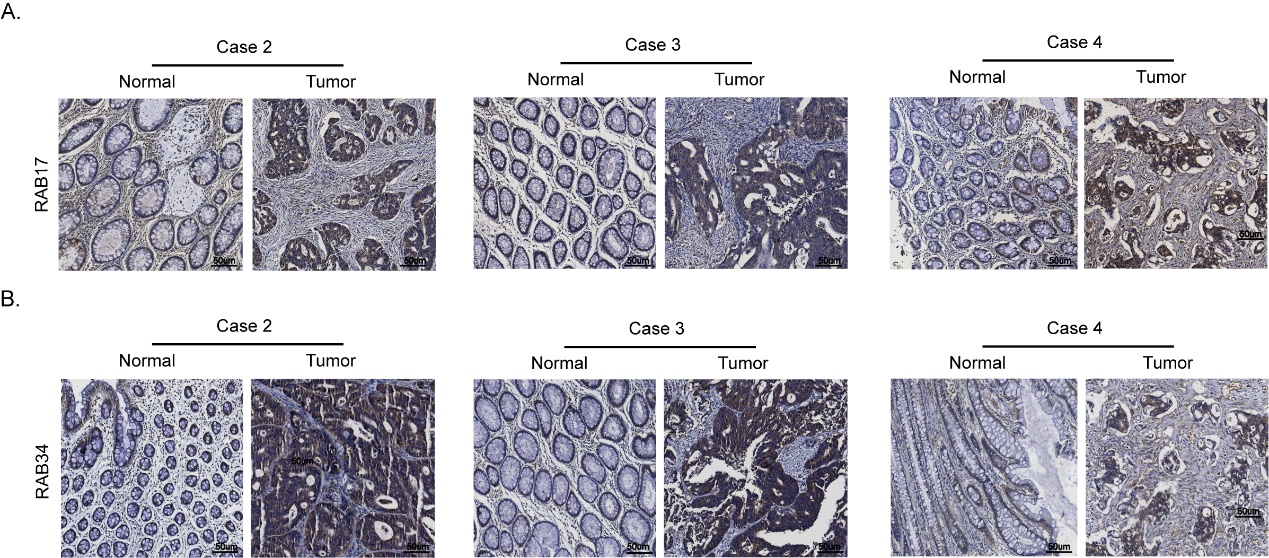
**
