# Supplementary material for: The Role of RAB GTPases and Its Potential in Predicting Immunotherapy Response and Prognosis in Colorectal Cancer
Source: Front Genet. 2022 Jan 28;13:828373. doi: 10.3389/fgene.2022.828373 (PMC8833848; doi:10.3389/fgene.2022.828373)
Supplement: Supplementary file 2 [file DataSheet2.ZIP › Supplementary Tables/Supplementary Table 10. Univariate Cox regression analysis of RAB34 mRNA expression in CRC patients from TCGA.docx]

**Supplementary Table 10**. Univariate Cox regression analysis of RAB34 mRNA expression in CRC patients from TCGA.

OS DFS

Variable HR (95%CI) P value HR (95%CI) P value

RAB34 expression

(high vs. low) 1.808 (1.096-2.982) 0.020* 1.989 (1.199-3.302) 0.008**

Gender (female vs. 0.624 (0.375-1.039) 0.070 0.777 (0.474-1.274) 0.317

male)

Median age (<=68

years vs . >68 years) 0.539 (0.327-0.890) 0.016* 1.319 (0.802-2.171) 0.275

pT stage (T3+T4 vs. 2.672 (1.066-6.698) 0.036* 2.043 (0.928-4.500) 0.076

T1+T2)

pN stage (N2 vs. 3.125 (1.874-5.211) 0.001*** 2.324 (1.343-4.021) 0.003**

T0+N1)

pM stage (M1 vs. M0) 4.928 (2.943-8.251) 0.001*** 3.495(1.969-6.205) 0.001***

Clinical stage (I+II vs. 0.308 (0.184-0.516) 0.001*** 0.478 (0.293-0.781) 0.003**

III+IV)

MSI (MSI-H vs. MSS 0.524 (0.250-1.099) 0.087 0.728 (0.380-1.395) 0.339

+MSI-L)

*P< 0.05, **P< 0.01, ***P< 0.001
