# Supplementary material for: The Role of RAB GTPases and Its Potential in Predicting Immunotherapy Response and Prognosis in Colorectal Cancer
Source: Front Genet. 2022 Jan 28;13:828373. doi: 10.3389/fgene.2022.828373 (PMC8833848; doi:10.3389/fgene.2022.828373)
Supplement: Supplementary file 2 [file DataSheet2.ZIP › Supplementary Tables/Supplementary Table 7. Correlation between expression of RAB17 and clinicopathological features for CRC patients.docx]

**Supplementary Table 7.** Correlation between expression of RAB17 and clinicopathological features for CRC patients.

| Variable | low RAB17 | high RAB17 | P value |  |
| --- | --- | --- | --- | --- |
|  | (n=280) | (n=174) |  |  |
| Gender |  |  | 0.668 |  |
| Female | 131 (46.8%) | 85 (48.9%) |  |  |
| Male | 149 (53.2%) | 89 (51.1%) |  |  |
| Median age |  |  | 0.602 |  |
| <=68 years | 141 (50.4%) | 92 (52.9%) |  |  |
| >68 years | 139 (49.6%) | 82 (47.1%) |  |  |
| pT staus |  |  | 0.054 |  |
| T1+T2 | 68 (24.3%) | 29 (16.7%) |  |  |
| T3+T4 | 212 (75.7%) | 145 (83.3%) |  |  |
| pN staus |  |  | 0.035* |  |
| N0+N1 | 243 (86.8%) | 138 (79.3%) |  |  |
| N2 | 37 (13.2%) | 36 (20.7%) |  |  |
| pM staus |  |  | 0.017* |  |
| M0 | 248 (88.6%) | 140 (80.5%) |  |  |
| M1 | 32 (11.4%) | 34 (19.5%) |  |  |
| Clinical stage |  |  | 0.001* |  |
| I+II | 180 (64.3%) | 83 (47.7%) |  |  |
| III+IV | 100 (35.7%) | 91 (52.3%) |  |  |
| Anatomical origin |  |  |  |  |
| Colon | 228 (81.4%) | 138 (79.3%) | 0.579 |  |
| Rectum  MSI | 52 (18.6%) | 36 (20.7%) | 0.923 |  |
| SS+MS-L | 221 (78.9%) | 138 (79.3%) |  |  |
| MSI-H | 59 (21.1%) | 36 (20.7%) |  |  |

*P< 0.05, **P< 0.01, ***P< 0.001
