# Supplementary material for: The Role of RAB GTPases and Its Potential in Predicting Immunotherapy Response and Prognosis in Colorectal Cancer
Source: Front Genet. 2022 Jan 28;13:828373. doi: 10.3389/fgene.2022.828373 (PMC8833848; doi:10.3389/fgene.2022.828373)
Supplement: Supplementary file 2 [file DataSheet2.ZIP › Supplementary Tables/Supplementary Table 8. Correlation between expression of RAB34 and clinicopathological features for CRC patients.docx]

**Supplementary Table 8.** Correlation between expression of RAB34 and clinicopathological features for CRC patients.

Variable low RAB34 high RAB34 P value

(n=223) (n=231)

Gender 0.659

Female 105 (47.1%) 104 (45.0%)

Male 118 (52.9%) 127 (55.0%)

Median age 0.842

<=68 years 116 (52.0%) 118 (51.1%)

>68 years 107 (48.0%) 113 (48.9%)

pT staus 0.222

T1+T2 54 (24.2%) 45 (19.5%)

T3+T4 169 (75.8%) 186 (80.5%)

pN staus 0.045*

N0+N1 195 (87.4%) 186 (80.5%)

N2 28 (12.6%) 45 (19.5%)

pM staus 0.613

M0 192 (86.1%) 195 (84.4%)

M1 31 (13.9%) 36 (15.6%)

Clinical stage 0.801

I+II 131 (58.7%) 98 (42.4%)

III+IV 92 (41.3%) 133 (57.6%)

Anatomical origin

Colon 188 (84.3%) 188 (81.4%) 0.410

Rectum 35 (15.7%) 43 (18.6%)

MSI 0.056

MSS+MS-L 167 (74.9%) 190 (82.3%)

MSI-H 56 (25.1%) 41 (17.7%)

*P< 0.05, **P< 0.01, ***P< 0.001
