# Supplementary material for: The Role of RAB GTPases and Its Potential in Predicting Immunotherapy Response and Prognosis in Colorectal Cancer
Source: Front Genet. 2022 Jan 28;13:828373. doi: 10.3389/fgene.2022.828373 (PMC8833848; doi:10.3389/fgene.2022.828373)
Supplement: Supplementary file 2 [file DataSheet2.ZIP › Supplementary Tables/Supplementary Table 9. Univariate Cox regression analysis of RAB17 mRNA expression in CRC patients from TCGA.docx]

**Supplementary Table 9.** Univariate Cox regression analysis of RAB17 mRNA expression in CRC patients from TCGA.

OS DFS

Variable HR (95%CI) P value HR (95%CI) P value

| RAB17 expression |  | | | | |
| --- | --- | --- | --- | --- | --- |
| (high vs. low) | 1.761 (1.086-2.856) | 0.022* | 1.792 (1.074-2.989) | 0.025* |  |
| Gender (female vs. | 0.751 (0.457-1.232) | 0.257 | 0.714 (0.423-1.205) | 0.208 |  |
| male) |  |  |  |  |  |
| Median age (<=68 |  |  |  |  |  |
| years vs . >68 years) | 0.469 (0.282-0.779) | 0.003** | 1.117 (0.667-1.872) | 0.673 |  |
| pT stage (T3+T4 vs. | 1.965 (0.890-4.336) | 0.095 | 1.437 (0.701-2.945) | 0.322 |  |
| T1+T2) |  |  |  |  |  |
| pN stage (N2 vs. | 2.776 (1.659-4.645) | 0.001*** | 2.161 (1.212-3.856) | 0.009** |  |
| T0+N1) |  |  |  |  |  |
| pM stage (M1 vs.  M0) | 4.448 (2.675-7.397) | 0.001*** | 3.319 (1.807-6.098) | 0.001*** |  |
| Clinical stage (I+II vs. | 0.366 (0.221-0.605) | 0.001*** | 0.556 (0.332-0.929) | 0.025* |  |
| III+IV) |  |  |  |  |  |
| MSI (MSI-H vs. MSS | 1.102 (0.517-2.347) | 0.802 | 0.677 (0.332-1.379) | 0.282 |  |
| +MSI-L) |  |  |  |  |  |

*P< 0.05, **P< 0.01, ***P< 0.001
